# Multiple Lower Limb Fractures in a Child With Spinal Muscular Atrophy Type 2: A Preventable Complication

**DOI:** 10.7759/cureus.53065

**Published:** 2024-01-27

**Authors:** Mounika Reddy, Vishnupriya Ambadas, Madhusudan Samprathi, Deepak Kumar Maley, Abhishek J Arora

**Affiliations:** 1 Pediatrics, All India Institute of Medical Sciences Bibinagar, Hyderabad, IND; 2 Orthopedics, All India Institute of Medical Sciences Bibinagar, Hyderabad, IND; 3 Radiodiagnosis, All India Institute of Medical Sciences Bibinagar, Hyderabad, IND

**Keywords:** fragility fractures, developmental delay disorders, early risk assessment, bone mineral density, physiotherapy education, pediatric genetics, neuromuscular diseases

## Abstract

Patients with spinal muscular atrophy (SMA) are at risk of poor bone health and fractures. We report a child with SMA type 2, presenting with acute pain and swelling of both lower limbs following physiotherapy, and found to have multiple fractures in both lower limbs. Literature on fractures in children with SMA is limited. Awareness of risk assessment and appropriate preventive measures among healthcare providers caring for children with SMA is essential.

## Introduction

Spinal muscular atrophy (SMA) is an autosomal, recessive, progressive neuromuscular disorder. Despite being primarily recognized for its impact on motor function, it is associated with significant, yet under-recognized comorbidities, including poor bone health, a topic that remains inadequately explored in literature [1,2]. In this context, we present a case of a child diagnosed with SMA type 2 who experienced multiple lower limb fractures following physiotherapy. This case underscores the importance of understanding and addressing the risk of poor bone health and fractures in children with SMA, particularly among healthcare providers in the context of therapeutic interventions.

## Case presentation

**Figure 1:**
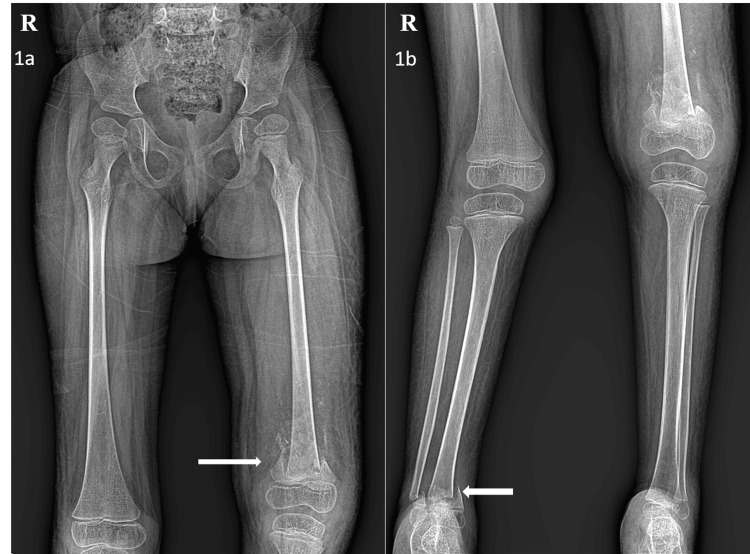
Antero-posterior view radiographs of the pelvis with both hips and the bilateral femur with knee joints (1a) and bilateral legs with ankle joints (1b), showing generalized decreased bone density with fracture of the distal metaphysis of the left femur with callus formation and fracture of the distal metaphyses of the right tibia and fibula (white arrows)

The patient, a four-and-a-half-year-old girl, presented with acute pain and swelling in both lower limbs for seven days after undergoing physiotherapy. She was diagnosed with SMA type 2 at two years of age when she first presented with delayed motor milestones, inability to walk, hypotonia, proximal muscle weakness, and areflexia. Genetic analysis by multiplex ligation-dependent probe amplification (MLPA) revealed a homozygous deletion of exons 7 and 8 in the survival motor neuron 1 (SMN1) gene. Hailing from a remote tribal area with limited access to healthcare, especially during the coronavirus disease 2019 (COVID-19) pandemic, she was not under regular follow-up. The family had approached a local practitioner ‘to gain power in the lower limbs’. There was no history of fever, falls, or intake of steroids. Examination revealed diffuse swelling of both lower limbs from thighs to ankles, mild local rise in temperature, exquisite tenderness, and pitting edema. The overlying skin appeared normal. Passive movements at both knees and ankles were restricted and painful and there were no hip contractures. She was thriving well with a weight of 13.6 kg (0 to -2 z score). Cellulitis and deep vein thrombosis were considered. Ultrasound showed diffuse subcutaneous edema in both lower limbs; venous Doppler was normal. The anteroposterior (AP) view of the X-ray of the pelvis and both lower limbs showed generalized, decreased bone density with comminuted transverse fracture of the distal metaphysis of the left femur with callus formation and fracture of distal metaphyses of the right tibia and fibula (Figure 1). There was no evidence of fractures anywhere else in the body, including upper limbs and vertebrae, or retinal hemorrhages to suggest abusive trauma. Hemoglobin was 7.9 g/dL, and total leucocyte count was 11,210/mm^3^; C-reactive protein, renal, and liver function tests were normal. Serum alkaline phosphatase was 250 IU/L (Table 1). Serum vitamin D levels and bone densitometry tests could not be done. Conservative management with analgesia and cast immobilization followed by early mobilization was initiated.

**Table 1:** Laboratory parameters of the index patient

Parameter	Normal value	Index patient
Hemoglobin (g/dL)	11.5-15.5	7.9
Total leucocyte count (x10^3^/µL)	3.50-10.00	11.21
Platelet count (x10^3^/µL)	150-400	598
Urea (mg/dL)	19-45	17
Creatinine (mg/dL)	0.7-1.3	0.1
Total bilirubin (mg/dL)	0-1	1.0
Total protein (g/dL)	6.4-8.3	6.7
Serum albumin (g/dL)	3.5-5.2	3.8
Aspartate transaminase (U/L)	<35	32
Alanine transaminase (U/L)	<45	15
Alkaline phosphatase (U/L)	42-128	250
C-reactive protein (mg/L)	<6	Negative

## Discussion

SMA, an autosomal recessive neuromuscular disorder characterized by progressive degeneration of lower motor neurons in the spinal cord, is due to homozygous loss of function mutations of the SMN1 gene on chromosome 5q. With an incidence of approximately 1 in 6,000-11,000 live births, it is a leading genetic cause of death among young children [2]. Patients with SMA type 2 have normal psychomotor development until six to eight months of age, with onset of weakness and delayed motor skills by 18 months; they never stand or walk independently [1,2].

Children with SMA, regardless of its type and severity, face a heightened risk of poor bone health characterized by low bone mineral density (BMD) and susceptibility to fragility fractures, often with minimal trauma. Predisposing factors include lack of ambulation and physical activity, reduced mechanical loading forces on the developing bone due to poor muscle mass and function, increased osteoclast activity and bone resorption associated with SMN1 mutation, and vitamin D deficiency [2]. There is limited literature on fractures in children with SMA. Recent retrospective studies suggest that the prevalence varies widely and increases with age and SMA severity, with the distal femur and vertebrae being the most common sites of fractures. Compared to reports from Europe and America, Chinese children with SMA had relatively fewer fractures, possibly due to decreased participation in rehabilitation and outdoor activities [3-5]. There are no published data on bone health and fractures in Indian patients with SMA.

Our patient had multiple fractures in various stages of healing involving both lower limbs. Considering the timing and severity of the fractures, it is likely that they were caused by inappropriate physiotherapy, and not by spontaneous event or minor trauma, irrespective of bone health status. This raises concerns about the appropriateness, safety, and potential impact of therapeutic interventions in SMA patients, considering their poor bone health. We found only one report of fractures following physiotherapy in the literature, which described two cases of periprosthetic fracture in adults who underwent hip arthroplasty [6]. Limb swelling following physiotherapy in children with SMA may be due to skin and soft tissue infections like cellulitis, muscle strain, deep venous thrombosis, lymphoedema, bone fractures, or rarely, an allergic reaction to the equipment or materials used during therapy. Fractures can worsen the already limited mobility and contractures in these patients. Given their fragile bones, respiratory problems, and anesthesia risk, nonoperative management with early mobilization is preferred in these patients as exemplified by the index patient [2].

As novel therapies and advances in supportive care improve the overall survival and quality of life for SMA patients, it becomes imperative to address the multifaced aspects of their health. Physiotherapy is recommended to maintain motor function and delay the progression of contractures [2]. Providing comprehensive multidisciplinary care in low-middle-income countries is challenging due to poor awareness among healthcare providers and families, limited availability of specialized staff and infrastructure, poor access to quality services, and inadequate funding. Healthcare providers caring for children with SMA must recognize the elevated risk of fractures in these patients, evaluate them judiciously, and exercise caution in physiotherapy and caregiving practices. Proactive management of bone health including measures to promote function and mobility, vitamin D and calcium supplementation, and annual monitoring of BMD is essential for a holistic approach to the care of children with SMA [2].

## Conclusions

We highlight the under-recognized comorbidity of poor bone health in a child with SMA, highlighting the intricate interplay between neurological disorders, bone health, and healthcare accessibility. It underscores the need for heightened awareness, proactive management, and a cautious approach to physiotherapy and caregiving practices in these patients.

## References

[1] Mercuri E, Sumner CJ, Muntoni F, Darras BT, Finkel RS (2022). Spinal muscular atrophy. Nat Rev Dis Primers.

[2] Mercuri E, Finkel RS, Muntoni F (2018). Diagnosis and management of spinal muscular atrophy: part 1: recommendations for diagnosis, rehabilitation, orthopedic and nutritional care. Neuromuscul Disord.

[3] Vai S, Bianchi ML, Moroni I (2015). Bone and spinal muscular atrophy. Bone.

[4] Wasserman HM, Hornung LN, Stenger PJ (2017). Low bone mineral density and fractures are highly prevalent in pediatric patients with spinal muscular atrophy regardless of disease severity. Neuromuscul Disord.

[5] Peng X, Qu Y, Li X, Liu J, Shan X, Wang J, Song F (2021). Bone mineral density and its influencing factors in Chinese children with spinal muscular atrophy types 2 and 3. BMC Musculoskelet Disord.

[6] Judkins TR, Dayton MR (2010). femoral neck fracture during physical therapy following surface replacement arthroplasty: a preventable complication? A case report. Patient Saf Surg.

